# Increasing the Chemical‐Shift Dispersion of Unstructured Proteins with a Covalent Lanthanide Shift Reagent

**DOI:** 10.1002/anie.201607261

**Published:** 2016-10-20

**Authors:** Christoph Göbl, Moritz Resch, Madeleine Strickland, Christoph Hartlmüller, Martin Viertler, Nico Tjandra, Tobias Madl

**Affiliations:** ^1^ Center for Integrated Protein Science MunichTechnische Universität MünchenDepartment of ChemistryLichtenbergstraße 485748GarchingGermany; ^2^ Institute of Structural BiologyHelmholtz Zentrum MünchenIngolstädter Landstr. 185764NeuherbergGermany; ^3^ Institute of Molecular Biology & BiochemistryCenter of Molecular MedicineMedical University of Graz8010GrazAustria; ^4^ Laboratory of Structural Biophysics Biochemistry and Biophysics CenterNational Heart, Lung, and Blood InstituteNational Institutes of HealthBuilding 50BethesdaMD20814USA

**Keywords:** chemical-shift dispersion, intrinsically disordered proteins, lanthanides, NMR, pseudo-contact shifts

## Abstract

The study of intrinsically disordered proteins (IDPs) by NMR often suffers from highly overlapped resonances that prevent unambiguous chemical‐shift assignments, and data analysis that relies on well‐separated resonances. We present a covalent paramagnetic lanthanide‐binding tag (LBT) for increasing the chemical‐shift dispersion and facilitating the chemical‐shift assignment of challenging, repeat‐containing IDPs. Linkage of the DOTA‐based LBT to a cysteine residue induces pseudo‐contact shifts (PCS) for resonances more than 20 residues from the spin‐labeling site. This leads to increased chemical‐shift dispersion and decreased signal overlap, thereby greatly facilitating chemical‐shift assignment. This approach is applicable to IDPs of varying sizes and complexity, and is particularly helpful for repeat‐containing IDPs and low‐complexity regions. This results in improved efficiency for IDP analysis and binding studies.

The well‐established structure–function paradigm has been challenged by the discovery of intrinsically disordered functional proteins (IDPs).^1^ Although lacking stable secondary or tertiary structure elements, this large class of proteins plays a crucial role in various cellular processes.^2^ It is suggested that about 40 % of all proteins have disordered regions of 40 or more residues, with many proteins existing solely in the unfolded state.^3^

NMR spectroscopy is a well‐suited method for studying the residual structure, dynamics, and interactions of IDPs with atomic resolution under near‐native conditions.^4^ The key step for these studies is the assignment of NMR resonances, which is particularly challenging for IDPs owing to poor chemical‐shift dispersion, which results in severe spectral overlap. This is due to a lack of well‐defined hydrogen bonds and a missing hydrophobic core with aromatic contributions.^5^ To reduce spectral overlap, several approaches have been proposed. In the “divide and conquer” approach, chemical‐shift assignments of shorter constructs obtained by standard NMR experiments are transferred to the full‐length protein.^6^ To assign chemical shifts of full‐length unstructured proteins, experiments have been proposed that make use of the favorable relaxation properties of IDPs in high‐dimensional experiments (up to 7D).^7^ These experiments reduce resonance overlap and can potentially provide unambiguous chemical‐shift assignments. An alternative strategy is the direct detection of heteronuclei, using the beneficial chemical‐shift dispersion of ^13^C^8^ or the increased resolution of direct detection of ^15^N.^9^ These approaches can be implemented easily with nonuniform sampling combined with non‐Fourier signal processing methods and fast‐pulsing techniques in order to reduce measurement time.^10^

Nevertheless, even providing that complete chemical‐shift assignments can be obtained, missing chemical‐shift dispersion in 2D NMR experiments complicates standard NMR approaches, including chemical‐shift perturbation mapping of protein–ligand interactions and studies of protein dynamics. In particular, unstructured low‐complexity regions display strong signal overlap due to their lack of sequence variation and repetitive nature.^11^

Herein, we present a simple and straightforward approach to increase the chemical‐shift dispersion of NMR resonances of an IDP and facilitate NMR chemical‐shift assignment when using standard methods. Our approach is based on the introduction of a rigid lanthanide‐chelating tag at a cysteine site. We demonstrate that this leads to enhanced chemical‐shift dispersion through a pseudo‐contact chemical shift (PCS) contribution for resonances up to 26 residues from the tagging site.

A large number of paramagnetic tags have been developed to exploit their effects on biomolecules.^12^ Lanthanides have been found to be particularly useful because the variations in their susceptibility tensors allow tuning of the desired effects through choosing a particular lanthanide.^13^ Cyclic polyamines have been shown to provide high lanthanide binding affinities with reasonable rigidity to minimize conformational averaging of the observed paramagnetic effects. Nevertheless, some tags can adopt different isomers after complex formation, which leads to the observation of multiple NMR signals. Various chemical modifications have been suggested to control the isomerization of the chelate.^14^

We decided to use a DOTA‐M8 ytterbium tag, here denoted as Yb‐M8 (DOTA=1,4,7,10‐tetraazacyclododecane‐1,4,7,10‐tetraacetic acid).^15^ This lanthanide chelator has the advantage of being rigid, which strongly increases the magnitude of the PCS. For most lanthanide ions, the Ln‐M8 tag shows two different conformations, which leads to a doubling of resonances. In contrast, ytterbium was shown to populate mainly one observable conformation.^16^ Furthermore, ytterbium causes moderate PCS and reasonable relaxation‐enhancement effects, such that all residues close to the tagging site can still be observed.

To demonstrate the suitability of our approach we used the intrinsically disordered arginine‐glycine‐glycine (RGG)‐repeat‐rich C‐terminal region of the protein “fused in sarcoma” (FUS, residues 454–526). FUS is a nuclear DNA/RNA‐binding protein that is the pathological hallmark protein in abnormal cytoplasmic and nuclear inclusions in some cases of frontotemporal lobar degeneration (FTLD) and amyotrophic lateral sclerosis (ALS).^17^ It contains two structured nucleotide‐binding domains and several disordered regions. The disordered C‐terminal RGG‐rich region has been identified as an arginine methylation site that is involved in nuclear transport and the formation of stress granules.^18^ This region harbors key interaction sites for several ligands, including the nuclear import factor transportin‐1 and G‐quadruplex RNAs.^18^, ^19^ The 36‐residue RGG‐rich region (472–505) contains 18 glycines and 10 arginines (Figure S1a in the Supporting Information) and lacks stable secondary structure. The NMR chemical shifts are challenging to assign because most of the ^1^H, ^15^N, and ^13^C resonances show severe overlap. By using a conventional NMR chemical‐shift assignment approach, we were able to unambiguously assign only a small fraction of the ^1^H and ^15^N backbone resonances (16 out of 36). To enhance the chemical‐shift dispersion of this region, we generated two single‐point cysteine mutants (D470C and D502C) at the N‐terminal and C‐terminal site of the RGG region and covalently attached the M8 lanthanide‐binding tags.

We found that this tag is still rigid enough when bound to the IDP to generate substantial PCS effects. Figure 1 a displays the effect of the lanthanide tag on the ^1^H^15^N‐HSQC spectrum of FUS. A large PCS effect can be observed in the surrounding resonances up to a maximum shift of 2.0 ppm in the amide ^1^H dimension (Figure 1 b). Chemical‐shift changes were detected for a number of amide resonances and a difference of 0.014 ppm was found for Gly496, which is 26 residues away from the labeled Cys470, corresponding to half of the full‐width half‐maximum of the peak (0.029 ppm in the ^1^H dimension). For position 470, the PCS effect was strongest for the C‐terminal part of the sequence and showed chemical‐shift changes inversely proportional to distance from the labeled cysteine residue. In contrast, for position 502, the PCS was higher for the N‐terminal region, meaning that for both tags, the PCS was stronger towards the center of the protein sequence and decayed more rapidly towards the termini. This highlights the sensitivity of the introduced tag to intrinsic protein dynamics. As a control, a diamagnetic sample was prepared by using the lutetium‐bound version of the tag positioned at C470. This tag yielded only small chemical shift changes in neighboring residues in the ^1^H^15^N spectra (Figure S2), which demonstrates that the observed effects originate from the paramagnetic ytterbium.

**Figure 1:**
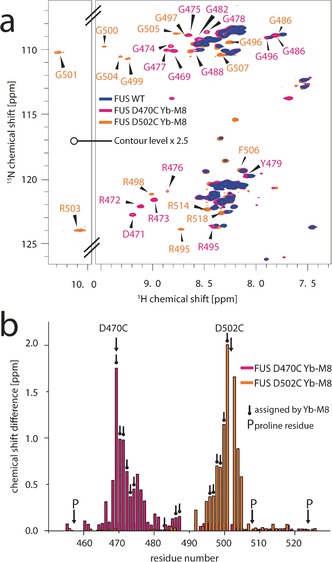
Spectral changes induced by the Yb‐M8 tag for FUS. a) ^1^H^15^N‐HSQC spectra of the reference (blue) and the Yb‐M8‐labeled (D470C magenta, D502C orange) spectrum of FUS(454–526). Each protein concentration is 200 μm and data were acquired with the same experimental time (8 scans, 128 complex data points, 1 s interscan delay) on a 900 MHz NMR spectrometer. b) ^1^H chemical‐shift differences of assigned residues between the two spectra. Peaks that could only be assigned in the Yb‐M8‐labeled samples are marked. Reference chemical shifts for these residues were determined by using an online prediction tool that performs sequence, temperature, and pH correction.^21^ Proline positions are marked with P.

Remote backbone residues and aromatic side chains (Figure S3) remain unaffected. After introducing the Yb‐M8 tag, the high dispersion allowed assignment of the shifted residues using standard CBCA(CO)NH and HNCACB NMR experiments (Figure S1b).^20^ The favorable relaxation properties of the introduced tag also allowed observation of the Cα/Cβ Cys470 and Cys502 resonances (Figure S1b). To further quantify the effect of the Yb‐M8 tag on the relaxation properties of FUS, we recorded longitudinal (R1) and transverse (R2 or R1ρ) amide proton and nitrogen relaxation rates for the Yb‐M8‐labeled and unlabeled protein (Figure S4). The relaxation rates were increased close to the tagging site (i.e. up to a 3.25 times higher proton R2 for G469 next to C470), and unaffected for residues with increasing distance from the tag, as expected for a non‐interfering modification. This shows that high‐quality NMR spectra can be obtained for the Yb‐M8 tag even for residues close to the tagging site, which are often not available when using other paramagnetic tags.

To further test the general applicability of the M8‐tag, we attached the Yb‐M8 label to the unstructured N‐terminal region of Lymphoid enhancer‐binding factor 1 (Lef‐1, residues 1–101), a key transcription factor of the WNT‐signaling pathway. The N‐terminal site includes a low‐complexity region with 8 consecutive glycine residues (ranging from residues 6 to 13) that appear in a single cluster in the ^1^H^15^N‐HSQC spectrum and are unassignable using conventional techniques. After attachment of the Yb‐M8 tag to the native cysteine, all of the glycine residues are well separated and can easily be assigned. Figure 2 a shows an overlay of the ^1^H^15^N‐HSQC for the reference and Yb‐M8‐labeled samples. The histogram (Figure 2 b) shows the ^1^H chemical‐shift differences; the largest chemical shift change was observed for Cys18 (1.2 ppm). There are two proline residues close to the label at positions *i*−3 and *i*+7, which clearly reduced the PCS of the neighboring residues through *cis*–*trans* racemization. Interestingly, although the N‐terminal PCS are all positive, as observed for both FUS mutants, the C‐terminal resonances show negative PCS values. The diamagnetic Lu‐M8‐tagged sample showed only small changes in the close vicinity of the labeled cysteine residue (Figure S5). Again, R_1_ and R_2_^15^N relaxation data (Figure S6) and chemical‐shift positions of remote residues do not indicate any additional intramolecular changes in the dynamic behavior of the protein. For the Lef‐1 sample, peaks outside the low‐complexity region are better resolved than in the FUS spectra. Direct comparison of the ^1^H^15^N‐HSQC peak intensities shows that the proximal resonances (residues 14–26, with the Yb‐M8 tag attached to Cys18) display a reduced intensity (Figure S7). The lowest intensity found is about 25 % of the unlabeled tag, which highlights the tolerable paramagnetic relaxation enhancement (PRE) effect of the Yb‐M8 label.

**Figure 2:**
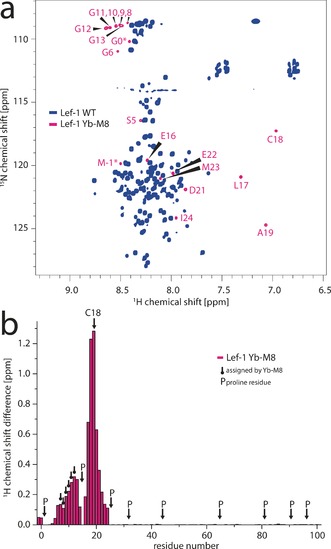
Spectral changes induced by the Yb‐M8 tag for Lef‐1(1–101). a) ^1^H^15^N‐HSQC spectra of the reference (blue) and the Yb‐M8‐labeled (pink) sample. Each protein concentration is 200 μm and data were acquired with the same experimental time (8 scans, 128 complex data points, 1 s inter‐scan delay) on a 900 MHz NMR spectrometer. Residues marked with (*) are part of a protease cleavage site (see the Supporting Information). b) Absolute values of ^1^H chemical‐shift differences of assigned residues between the two spectra. Peaks that could only be assigned in the Yb‐M8‐labeled samples are marked. Proline positions are marked with P.

Introduction of the Yb‐M8 tag is a general approach that can alleviate the problem of chemical‐shift degeneracy in IDPs, and in repeat regions in particular. To demonstrate the practicability of our approach, we titrated the D470C Yb‐M8‐labeled version of FUS with a biological interaction partner, the 110 kDa nuclear import protein transportin‐1. So far, only the interaction with the C‐terminal domain of FUS has been structurally characterized,^22^ but recent findings have suggested a role of the preceding RGG‐rich region.^18^, ^23^ The introduction of the tag allowed observation of spectral changes of this repetitive protein sequence with single‐residue resolution. This region has previously been too overlapped for unique assignments, but now it could be demonstrated that the RGG‐rich region is involved in transportin‐1 interaction (Figure S8).

Although cysteine residues are typically underrepresented in unstructured protein regions, they can easily be engineered into the protein sequence so that the tag can be incorporated at the site of interest.^24^ If necessary, multiple sites can be mutated and tagged to resolve longer protein sequences. The enhanced chemical‐shift dispersion greatly simplifies the NMR studies of both protein interactions and dynamics when the Yb‐M8 tag is attached. In particular for studying protein–ligand interactions, the 1/*r* ^3^ long‐range distance dependence of the PCS effect is of great benefit. Clearly, and as for any other commonly used tag, it must be ensured that introduction of the Yb‐M8 tag does not interfere with native protein interactions. Nevertheless, the Yb‐M8 tag is highly polar and water soluble compared to the frequently used hydrophobic aminoxyl tags.^12^, ^25^ In summary, we present a simple and straightforward approach to resolve severe chemical‐shift overlap in disordered proteins by exploiting the substantial PCS induced by the ytterbium‐M8 tag. The enhanced chemical‐shift dispersion and long‐range PCS effects provide a well‐suited starting point for studying the protein–ligand interactions and dynamics of IDPs by NMR. Given that IDPs represent a large fraction of the proteome, are often rich in repeat regions, and act as key interaction hubs with cofactors, our approach will greatly facilitate resolution of the molecular mechanisms of these intricate, highly biologically relevant interaction networks.

## Supporting information

As a service to our authors and readers, this journal provides supporting information supplied by the authors. Such materials are peer reviewed and may be re‐organized for online delivery, but are not copy‐edited or typeset. Technical support issues arising from supporting information (other than missing files) should be addressed to the authors.

Supplementary

## References

[1] H. J. Dyson , P. E. Wright , Nat. Rev. Mol. Cell Biol. 2005, 6, 197–208. 15738986 10.1038/nrm1589

[2a] F.-X. Theillet , A. Binolfi , T. Frembgen-Kesner , K. Hingorani , M. Sarkar , C. Kyne , C. Li , P. B. Crowley , L. Gierasch , G. J. Pielak , A. H. Elcock , A. Gershenson , P. Selenko , Chem. Rev. 2014, 114, 6661–6714; 24901537 10.1021/cr400695p PMC4095937

[2b] P. E. Wright , H. J. Dyson , Nat. Rev. Mol. Cell Biol. 2015, 16, 18–29; 25531225 10.1038/nrm3920 PMC4405151

[2c] R. van der Lee , M. Buljan , B. Lang , R. J. Weatheritt , G. W. Daughdrill , A. K. Dunker , M. Fuxreiter , J. Gough , J. Gsponer , D. T. Jones , P. M. Kim , R. W. Kriwacki , C. J. Oldfield , R. V. Pappu , P. Tompa , V. N. Uversky , P. E. Wright , M. M. Babu , Chem. Rev. 2014, 114, 6589–6631; 24773235 10.1021/cr400525m PMC4095912

[2d] V. N. Uversky , V. Davé , L. M. Iakoucheva , P. Malaney , S. J. Metallo , R. R. Pathak , A. C. Joerger , Chem. Rev. 2014, 114, 6844–6879. 24830552 10.1021/cr400713r PMC4100540

[3a] P. Tompa , Trends Biochem. Sci. 2002, 27, 527–533; 12368089 10.1016/s0968-0004(02)02169-2

[3b] P. Romero , Z. Obradovic , C. R. Kissinger , J. E. Villafranca , S. Guilliot , E. Garner , A. K. Dunker , Pac. Symp. Biocomput. 1998, 3, 437–448. 9697202

[4a] M. Kjaergaard , F. M. Poulsen , Prog. Nucl. Magn. Reson. Spectrosc. 2012, 60, 42–51; 22293398 10.1016/j.pnmrs.2011.10.001

[4b] I. C. Felli , R. Pierattelli , Intrinsically Disordered Proteins Studied by NMR Spectroscopy (Eds. I. C. Felli, R. Pierattelli), Springer International Publishing, Switzerland, 2015;

[4c] H. J. Dyson , P. E. Wright , Chem. Rev. 2004, 104, 3607–3622; 15303830 10.1021/cr030403s

[4d] S. Kosol , S. Contreras-Martos , C. Cedeño , P. Tompa , Molecules 2013, 18, 10802; 24008243 10.3390/molecules180910802 PMC6269831

[4e] M. R. Jensen , M. Zweckstetter , J.-r. Huang , M. Blackledge , Chem. Rev. 2014, 114, 6632–6660. 24725176 10.1021/cr400688u

[5] S. Schwarzinger , G. J. A. Kroon , T. R. Foss , J. Chung , P. E. Wright , H. J. Dyson , J. Am. Chem. Soc. 2001, 123, 2970–2978. 11457007 10.1021/ja003760i

[6] M. D. Mukrasch , S. Bibow , J. Korukottu , S. Jeganathan , J. Biernat , C. Griesinger , E. Mandelkow , M. Zweckstetter , PLoS Biol. 2009, 7, e1000034. 10.1371/journal.pbio.1000034 PMC2642882 19226187

[7a] A. Zawadzka-Kazimierczuk , W. Koźmiński , H. Šanderová , L. Krásný , J. Biomol. NMR 2012, 52, 329–337; 22350953 10.1007/s10858-012-9613-x PMC3315646

[7b] V. Motáčková , J. Nováček , A. Zawadzka-Kazimierczuk , K. Kazimierczuk , L. Žídek , H. Šanderová , L. Krásný , W. Koźmiński , V. Sklenář , J. Biomol. NMR 2010, 48, 169–177; 20890634 10.1007/s10858-010-9447-3 PMC2966349

[7c] R. L. Narayanan , U. H. N. Dürr , S. Bibow , J. Biernat , E. Mandelkow , M. Zweckstetter , J. Am. Chem. Soc. 2010, 132, 11906–11907; 20687558 10.1021/ja105657f

[7d] A. Piai , T. Hošek , L. Gonnelli , A. Zawadzka-Kazimierczuk , W. Koźmiński , B. Brutscher , W. Bermel , R. Pierattelli , I. Felli , J. Biomol. NMR 2014, 60, 209–218; 25326659 10.1007/s10858-014-9867-6

[7e] I. Bagai , S. Ragsdale , E. P. Zuiderweg , J. Biomol. NMR 2011, 49, 69–74. 21190062 10.1007/s10858-010-9465-1 PMC3091507

[8a] T. E. Machonkin , W. M. Westler , J. L. Markley , J. Am. Chem. Soc. 2004, 126, 5413–5426; 15113213 10.1021/ja037077i

[8b] W. Bermel , M. Bruix , I. Felli , V. M. V. Kumar , R. Pierattelli , S. Serrano , J. Biomol. NMR 2013, 55, 231–237; 23314728 10.1007/s10858-013-9704-3

[8c] I. C. Felli , R. Pierattelli , IUBMB Life 2012, 64, 473–481; 22556167 10.1002/iub.1045

[8d] W. Bermel , I. Bertini , I. C. Felli , Y.-M. Lee , C. Luchinat , R. Pierattelli , J. Am. Chem. Soc. 2006, 128, 3918–3919; 16551093 10.1021/ja0582206

[8e] W. Bermel , I. C. Felli , R. Kümmerle , R. Pierattelli , Concepts Magn. Reson. Part A 2008, 32, 183–200.

[9] M. Gal , K. A. Edmonds , A. G. Milbradt , K. Takeuchi , G. Wagner , J. Biomol. NMR 2011, 51, 497–504. 22038648 10.1007/s10858-011-9580-7 PMC3338130

[10a] S. Żerko , W. Koźmiński , J. Biomol. NMR 2015, 63, 283–290; 26403428 10.1007/s10858-015-9987-7 PMC4642589

[10b] I. C. Felli , B. Brutscher , ChemPhysChem 2009, 10, 1356–1368; 19462391 10.1002/cphc.200900133

[10c] S. Hiller , C. Wasmer , G. Wider , K. Wüthrich , J. Am. Chem. Soc. 2007, 129, 10823–10828; 17691781 10.1021/ja072564+

[10d] S. Hiller , F. Fiorito , K. Wüthrich , G. Wider , Proc. Natl. Acad. Sci. USA 2005, 102, 10876–10881; 16043707 10.1073/pnas.0504818102 PMC1182451

[10e] M. Nowakowski , S. Saxena , J. Stanek , S. Żerko , W. Koźmiński , Prog. Nucl. Magn. Reson. Spectrosc. 2015, 90–91, 49–73; 10.1016/j.pnmrs.2015.07.001 26592945

[10f] M. Mobli , J. C. Hoch , Prog. Nucl. Magn. Reson. Spectrosc. 2014, 83, 21–41. 25456315 10.1016/j.pnmrs.2014.09.002 PMC5776146

[11a] A. Coletta , J. W. Pinney , D. Y. W. Solís , J. Marsh , S. R. Pettifer , T. K. Attwood , BMC Syst. Biol. 2010, 4, 43; 20385029 10.1186/1752-0509-4-43 PMC2873317

[11b] B. Kumari , R. Kumar , M. Kumar , Mol. BioSyst. 2015, 11, 585–594. 25468592 10.1039/c4mb00425f

[12] C. Göbl , T. Madl , B. Simon , M. Sattler , Prog. Nucl. Magn. Reson. Spectrosc. 2014, 80, 26–63. 24924266 10.1016/j.pnmrs.2014.05.003

[13] G. Otting , Annu. Rev. Biophys. 2010, 39, 387–405. 20462377 10.1146/annurev.biophys.093008.131321

[14a] B. Graham , C. T. Loh , J. D. Swarbrick , P. Ung , J. Shin , H. Yagi , X. Jia , S. Chhabra , N. Barlow , G. Pintacuda , T. Huber , G. Otting , Bioconjugate Chem. 2011, 22, 2118–2125; 10.1021/bc200353c 21877751

[14b] M. D. Vlasie , C. Comuzzi , A. M. C. H. van den Nieuwendijk , M. Prudêncio , M. Overhand , M. Ubbink , Chem. Eur. J. 2007, 13, 1715–1723; 17115462 10.1002/chem.200600916

[14c] P. H. J. Keizers , A. Saragliadis , Y. Hiruma , M. Overhand , M. Ubbink , J. Am. Chem. Soc. 2008, 130, 14802–14812. 18826316 10.1021/ja8054832

[15] D. Häussinger , J.-r. Huang , S. Grzesiek , J. Am. Chem. Soc. 2009, 131, 14761–14767. 19785413 10.1021/ja903233w

[16] A. C. L. Opina , M. Strickland , Y.-S. Lee , N. Tjandra , R. Andrew Byrd , R. E. Swenson , O. Vasalatiy , Dalton Trans. 2016, 45, 4673–4687. 26857249 10.1039/c5dt03210e PMC4807635

[17] E. Bentmann , C. Haass , D. Dormann , FEBS J. 2013, 280, 4348–4370. 23587065 10.1111/febs.12287

[18] D. Dormann , T. Madl , C. F. Valori , E. Bentmann , S. Tahirovic , C. Abou-Ajram , E. Kremmer , O. Ansorge , I. R. A. Mackenzie , M. Neumann , C. Haass , EMBO J. 2012, 31, 4258–4275. 22968170 10.1038/emboj.2012.261 PMC3501225

[19] N. Vasilyev , A. Polonskaia , J. C. Darnell , R. B. Darnell , D. J. Patel , A. Serganov , Proc. Natl. Acad. Sci. USA 2015, 112, E5391. 10.1073/pnas.1515737112 PMC4593078 26374839

[20] S. Grzesiek , A. Bax , J. Magn. Reson. 1992, 99, 201–207.

[21] National Institutes of Health, NIDDK, http://spin.niddk.nih.gov/bax/nmrserver/Poulsen_rc_CS (accessed January 14, 2016).

[22] Z. C. Zhang , Y. M. Chook , Proc. Natl. Acad. Sci. USA 2012, 109, 12017–12021. 22778397 10.1073/pnas.1207247109 PMC3409756

[23] M. Suárez-Calvet , M. Neumann , T. Arzberger , C. Abou-Ajram , E. Funk , H. Hartmann , D. Edbauer , E. Kremmer , C. Göbl , M. Resch , B. Bourgeois , T. Madl , S. Reber , D. Jutzi , M.-D. Ruepp , I. R. A. Mackenzie , O. Ansorge , D. Dormann , C. Haass , Acta Neuropathol. 2016, 131, 587–604. 26895297 10.1007/s00401-016-1544-2

[24] R. M. Williams , Z. Obradović , V. Mathura , W. Braun , E. C. Garner , J. Young , S. Takayama , C. J. Brown , A. K. Dunker , Pac. Symp. Biocomput. 2001, 6, 89–100. 10.1142/9789814447362_0010 11262981

[25] A. Bernini , V. Venditti , O. Spiga , N. Niccolai , Prog. Nucl. Magn. Reson. Spectrosc. 2009, 54, 278–289.

