# Supplementary material for: Increasing the Chemical‐Shift Dispersion of Unstructured Proteins with a Covalent Lanthanide Shift Reagent
Source: Angew Chem Int Ed Engl. 2016 Oct 20;55(47):14847–51. doi: 10.1002/anie.201607261 (PMC5146990; doi:10.1002/anie.201607261)
Supplement: Supplementary file 1 — Supplementary [file ANIE-55-14847-s001.pdf]

Angewandte Chemie
Eine Zeitschrift der Gesellschaft Deutscher Chemiker

Supporting Information

Increasing the Chemical-Shift Dispersion of Unstructured Proteins with a Covalent Lanthanide Shift Reagent

Christoph Göbl, Moritz Resch, Madeleine Strickland, Christoph Hartlmüller, Martin Viertler, Nico Tjandra, and Tobias Madl**

anie_201607261_sm_miscellaneous_information.pdf

Expression, purification and attachment of the M8 spin label tag of FUS (454-526, D470C and D502C) and Lef-1 (1-101)

The genes of FUS (residue no. 454-526 and harboring a D470C or D502C mutation, codon optimized for *Escherichia coli* (*E. coli*), GATC Biotech, Konstanz, Germany) and Lef-1 (residue no. 1-101, codon optimized for *Escherichia coli* (*E. coli*), ATG:biosynthetics GmbH, Merzhausen Germany) were cloned into a modified pET-M11 vector that contains an N-terminal His-tag followed by the Z domain of *Staphylococcal* protein A (58 residues) and a TEV (tobacco etch virus) cleavage site leaving three additional N-terminal amino acids (GAM) after cleavage. The expression vectors were transformed into *E. Coli* Bl21 (DE3) and cells were grown at 37°C in the presence of kanamycin. After inoculation of 100 ml of M9 minimal medium including uniformly ^{13}C labeled glucose (2.5 g per liter) and uniformly ^{15}N labeled ammonium chloride (1 g per liter), the cultures were grown overnight in a shaking incubator. Next morning, the cell suspension was diluted with 900 ml of the same medium and grown to an OD of 0.7 (FUS) and 0.8 (Lef-1) and protein synthesis was induced by addition of 1 ml of a 0.5 M IPTG (isopropyl-1-thio-D-galactopyranoside) stock solution to a final concentration of 0.5 mM. The culture was incubated overnight at 19°C (FUS) and 20°C (Lef-1) and harvested the next day. The cell pellets were re-suspended in 30 ml FUS urea purification buffer (8 M urea, 20 mM Tris, pH 8.0 and 20 mM imidazole) or Lef-1 urea purification buffer (6 M urea, 50 mM Tris, pH 8.0, 300 mM NaCl, 10 mM imidazole, 2 mM BME), sonicated, centrifuged at 25000 g for 20 min and applied to a Ni-NTA agarose (Qiagen) gravity column following the manufacturer's instruction. After washing of the column with 50 ml urea buffer, the buffer for FUS was exchanged to HEPES buffer (110 mM potassium acetate, 20 mM HEPES, pH 8.0, 2 mM β-mercaptoethanol (BME), 5% (v/v) glycerol and 20 mM imidazole). After urea buffer treatment of Lef-1, the protein was further washed with high salt buffer (20 ml of 50 mM Tris, pH 8.0, 1 M NaCl, 10 mM imidazole, 2 mM BME) and equilibrated with 20 ml Tris buffer (50 mM Tris, pH 8.0, 300 mM NaCl, 10 mM imidazole, 2 mM BME). The proteins were then eluted with the elution buffer ((50 mM Tris, pH 8.0, 300 mM NaCl, 200 mM imidazole, 2 mM BME) including 200 mM imidazole. FUS was concentrated to 5 ml in an Amicon Ultra-15 (Millipore, 3 kDa molecular weight cut-off) centrifugal filter unit to be applied to size exclusion chromatography. After loading on a HiLoad 16/600 Superdex 75 pg (GE Healthcare Life Sciences, 50 mM sodium phosphate, 500 mM NaCl, 2 mM BME, pH 6.0) the FUS-containing fractions were pooled and dialyzed over night at 4°C against HEPES buffer using a 2 kDa MWCO ZelluTrans V series membrane (Carl Roth) after addition of 200 µl of a 0.1 mg/ml His-tagged TEV protease solution. The next day, the solution was applied to a Ni-NTA agarose column to remove the cleaved tag, the TEV protease and uncleaved protein. The flow-through fraction was collected and split into two fractions. One fraction was buffer exchanged into NMR buffer (20 mM sodium phosphate buffer, 50 mM NaCl, 1 mM EDTA, pH 6.8, 1 mM DTT) to serve as the reference sample by using a HiPrep (26/10) desalting column (GE Healthcare Life Sciences). The other fraction was labeled with the ytterbium-M8 or lutetium-M8 tag[1] by first incubating with 1 mM TCEP for 30 minutes followed by buffer exchange into 10 mM sodium phosphate (pH 5.0) using the desalting column. Lef-1 was incubated with 200 µl of a 0.1 mg/ml 6xHis- tagged TEV protease solution after elution from the Ni-NTA resin and dialyzed over night at 4°C using a 2 kDa MWCO ZelluTrans V series membrane (Carl Roth) against Tris buffer. The next day, a heat-shock in boiling water was performed for 15 minutes and the protein solution was concentrated to 350 µM and frozen at -80°C for further usage. For the M8-attachment, the protein was thawed and applied to size exclusion chromatography like FUS, the running buffer was Tris buffer with 150 mM NaCl and incubated with 2 mM TCEP for 30 minutes. For the labeling reactions, the protein solutions were concentrated to 200 µM and combined with the three-fold molar excess of the tag. The reaction was finished after 15 minutes as monitored by NMR spectroscopy for FUS. The reaction solution was then buffer-exchanged into NMR buffer to remove the excess tag by a desalting step into NMR buffer lacking DTT for NMR data spectra acquisition. The NMR buffer for Lef-1 was 20 mM sodium phosphate, pH 6.5, 50 mM NaCl. The purification of the interaction partner transportin-1 was performed as described previously[2].

NMR data acquisition and analysis

The protein solutions (reference and ytterbium-M8 or lutetium-M8 spin labeled, 200 µM concentration) included 5 vol-% ^2H_2O and were measured at 298 K. All NMR experiments were performed on an AV900 MHz Bruker NMR spectrometer equipped with a cryogenic triple resonance probe with gradients in the z-direction, except the lutetium tag labeled sample, which was measured on an AV III 950 with the same hardware as the AV900. The sweep widths for ^{1}H dimensions were set to 14 ppm [carrier frequency (c.f.) of 4.7 ppm], for ^{15}N experiments to 20 ppm (c. f. of 117.5 ppm) and for ^{13}C carbonyl experiments to 17 ppm (c. f. of 176 ppm) and for Cα/Cβ carbon experiments to 65 ppm (c. f. of 42 ppm), respectively. Besides the ^{1}H^{15}N-HSQC spectrum, the following three-dimensional spectra were acquired for assignment of the FUS reference sample: HNCO, HN(CA)CO, HNCACB, CBCA(CO)NH, (H)CC(CO)NH-TOCSY (two experiments, with 11 and 22 ms mixing time, respectively), HN(CA)NNH and H(NCA)NNH[3]. For the M8 spin labeled protein, HNCO, HN(CA)CO, HNCACB and CBCA(CO)NH spectra were acquired. Lef-1 constructs (reference, Yb-M8 and Lu-M8 labeled) were assigned by an HNCACB and two HNCANNH spectra with evolution in the third dimension in ^{1}H and ^{15}N, respectively. The longitudinal relaxation data for the amide ^{1}H resonances was acquired by a modified ^{1}H^{15}N HSQC experiment as described[4] including a saturation block for the proton resonances in the beginning of the sequence followed by a recovery delay (0.01; 4; 0.02; 2.0; 0.038; 1.05; 0.07; 0.054; 0.14; 0.54 and 0.27 s) and WATERGATE type solvent suppression at the end of the sequence. Eleven two-dimensional data sets with different recovery delays were acquired in an interleaved manner where after each 4 accumulating scans the next delay was measured followed by TPPI receiver phase incrementation for quadrature detection in the indirect dimension. One recovery delay was measured twice for error estimation during the data fitting and a total of 128 complex data points was acquired. For measurement of the amide proton transverse relaxation time, a modified ^{1}H,^{15}N HSQC experiment was used as described[5] where after proton excitation a delay *t* was applied during which relaxation took place. During *t*, selective amide proton decoupling was applied by a selective 180° pulse (RE-BURP, 2.32 kHz band-width, 8.5 ppm center) in the middle of the delay flanked by ^{15}N decoupling (10; 100; 11; 67; 12; 46; 12; 46; 14; 32 and 18 ms delays). Water suppression was carried out by a WATERGATE scheme. After accumulation of 8 scans, the receiver phase was incremented for TPPI-type quadrature detection followed by incrementation of the delay t. A total of 120 complex data points were acquired in the indirect dimension and two delays were measured twice for error estimation. For the ^{15}N relaxation data, pulse sequences also included a WATERGATE scheme or water suppression. The longitudinal relaxation rate was measured by using delay times of 2764.8, 10.8, 172.8, 1382.4, 86.4, 1015.2, 172.8, 691.2, 345.6 and 259.2 ms for nitrogen magnetization decay and a 2 s inter-scan delay. The transverse relaxation rate data was measured using a 2 kHz spin lock pulse during the nitrogen relaxation period (length of delays for all samples was 5, 300, 50, 192, 12, 123, 12, 78, 32 and 400 ms) and a 2 s inter-scan delay. Offset correction for conversion of the T1ρ data and measurement of the heteronuclear NOE (interscan delay of 2 s) was performed as described elsewhere. [6]. All data were processed using NMRPipe[7], peak assignment and spectral visualization was performed using CCPNMR[8]. For analysis of the relaxation rates, peak intensities were first obtained using nmrglue[9] by averaging over all data points around the peak maxima in a 0.1/0.01 ppm (^{15}N/^{1}H) box of well-separated peaks. Using the SciPy python package, fitting and error estimation were carried out using a lab-written software. For analysis of longitudinal relaxation data, intensities were fitted to equation 1,

$$I = -I_0 \cdot e^{-R_1 \cdot t} + C \qquad (1)$$

where I is the intensity of the data point, I_0 is the maximum intensity, t is the recovery delay, C is the plateau of the fitting curve and R_1 is the longitudinal relaxation rate. The same procedure was applied for analysis of transverse relaxation rates and the obtained data was fitted to equation 2,

$$I = I_0 \cdot e^{-R_2 \cdot t} + D \qquad (2)$$

where I, I_0 and t are the same variables as in eq. 1, D is the baseline offset and R_2 is the transverse relaxation time.

To estimate the experimental error of the relaxation experiments, the differences in intensity in the duplicated spectra were calculated for all peaks. The differences d_i were then shifted to obtain a distribution centered on zero and the experimental error E_{exp} was obtained by calculating the standard deviation using equation 3,

$$E_{exp} = \sqrt{\frac{1}{2N} \cdot \sum_{i=1}^{N} d_i} \qquad (3)$$

where N is the number of peaks and d_i is the shifted difference of the duplicates of the i-th peak.

The errors of the fitted parameters were estimated using a Monte Carlo-like resampling approach with an ensemble size of 1000. To generate the ensemble, the peak intensities were randomly altered according to a normal distribution with a standard deviation of E_{exp}. Furthermore, a bootstrapping strategy was applied simultaneously by building new data sets, each having 27 data points that were randomly drawn from the data points. This ensemble was re-fitted using equation 1 or 2 in the case of longitudinal or transverse relaxation rate, respectively. The final fitting error of every parameter is obtained from the standard deviation over the 1000 fitting results

References

[1] a) D. Häussinger, J.-r. Huang, S. Grzesiek, *Journal of the American Chemical Society* **2009**, *131*, 14761-14767; b) A. C. L. Opina, M. Strickland, Y.-S. Lee, N. Tjandra, R. Andrew Byrd, R. E. Swenson, O. Vasalatiy, *Dalton Transactions* **2016**, *45*, 4673-4687.

[2] M. Suárez-Calvet, M. Neumann, T. Arzberger, C. Abou-Ajram, E. Funk, H. Hartmann, D. Edbauer, E. Kremmer, C. Göbl, M. Resch, B. Bourgeois, T. Madl, S. Reber, D. Jutzi, M.-D. Ruepp, I. R. A. Mackenzie, O. Ansorge, D. Dormann, C. Haass, *Acta Neuropathologica* **2016**, *131*, 587-604.

[3] R. Weisemann, H. Rüterjans, W. Bermel, *J Biomol NMR* **1993**, *3*, 113-120.

[4] a) T. Madl, T. Güttler, D. Görlich, M. Sattler, *Angewandte Chemie International Edition* **2011**, *50*, 3993-3997; b) H. G. Hocking, K. Zangger, T. Madl, *ChemPhysChem* **2013**, *14*, 3082-3094; c) C. Göbl, T. Madl, B. Simon, M. Sattler, *Progress in Nuclear Magnetic Resonance Spectroscopy* **2014**, *80*, 26-63.

[5] J. Iwahara, C. D. Schwieters, G. M. Clore, *Journal of the American Chemical Society* **2004**, *126*, 5879-5896.

[6] a) N.-A. Lakomek, J. Ying, A. Bax, *J Biomol NMR* **2012**, *53*, 209-221; b) J. W. Peng, V. Thanabal, G. Wagner, *Journal of Magnetic Resonance (1969)* **1991**, *94*, 82-100; c) A. G. Palmer, *Chemical Reviews* **2004**, *104*, 3623-3640.

[7] F. Delaglio, S. Grzesiek, G. Vuister, G. Zhu, J. Pfeifer, A. Bax, *J Biomol NMR* **1995**, *6*, 277-293.

[8] W. F. Vranken, W. Boucher, T. J. Stevens, R. H. Fogh, A. Pajon, M. Llinas, E. L. Ulrich, J. L. Markley, J. Ionides, E. D. Laue, *Proteins: Structure, Function, and Bioinformatics* **2005**, *59*, 687-696.

[9] J. Helmus, C. Jaroniec, *J Biomol NMR* **2013**, *55*, 355-367.

[10] J. Jeener, B. H. Meier, P. Bachmann, R. R. Ernst, *The Journal of Chemical Physics* **1979**, *71*, 4546-4553.

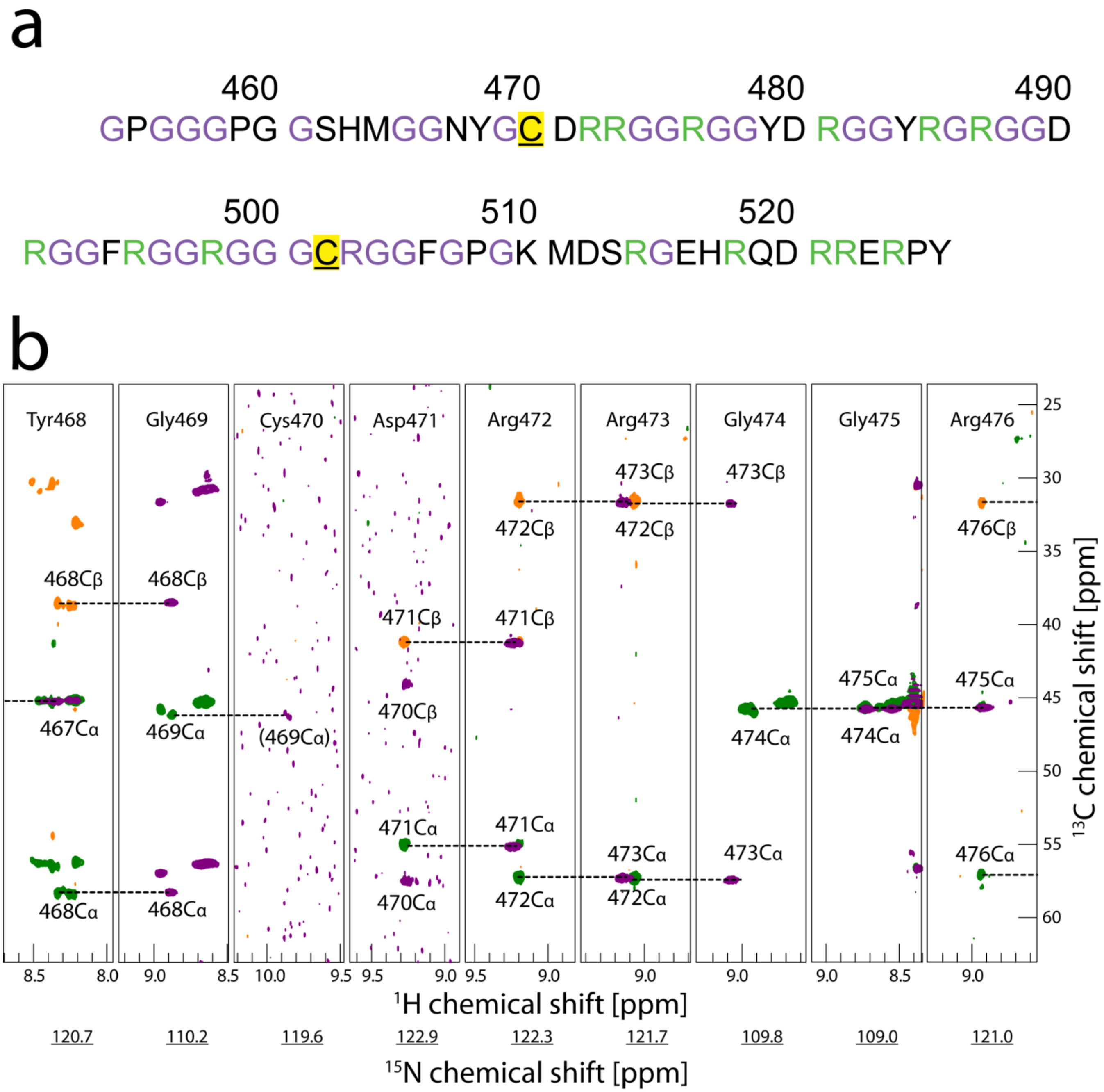

Figure S1. (a) Sequence of FUS (454-526) highlighting the positions of the introduced cysteine and glycine residues in purple and arginine residues in green, respectively. The Yb-M8 labeled constructs harbored one single mutation each. (b) Sequential walk for the assignment of the residues around the ytterbium-M8 labeled C470 residue. A HNCACB experiment shows Cα residues in green and Cβ residues in orange and an overlay of an CBCACONH experiment displays peaks in purple. The corresponding residues are indicated in the top of the panel and resonances are labeled, the nitrogen chemical shift is given in the bottom. Dashed lines indicate the sequential connections. Contour levels have been adjusted individually for representation purposes.

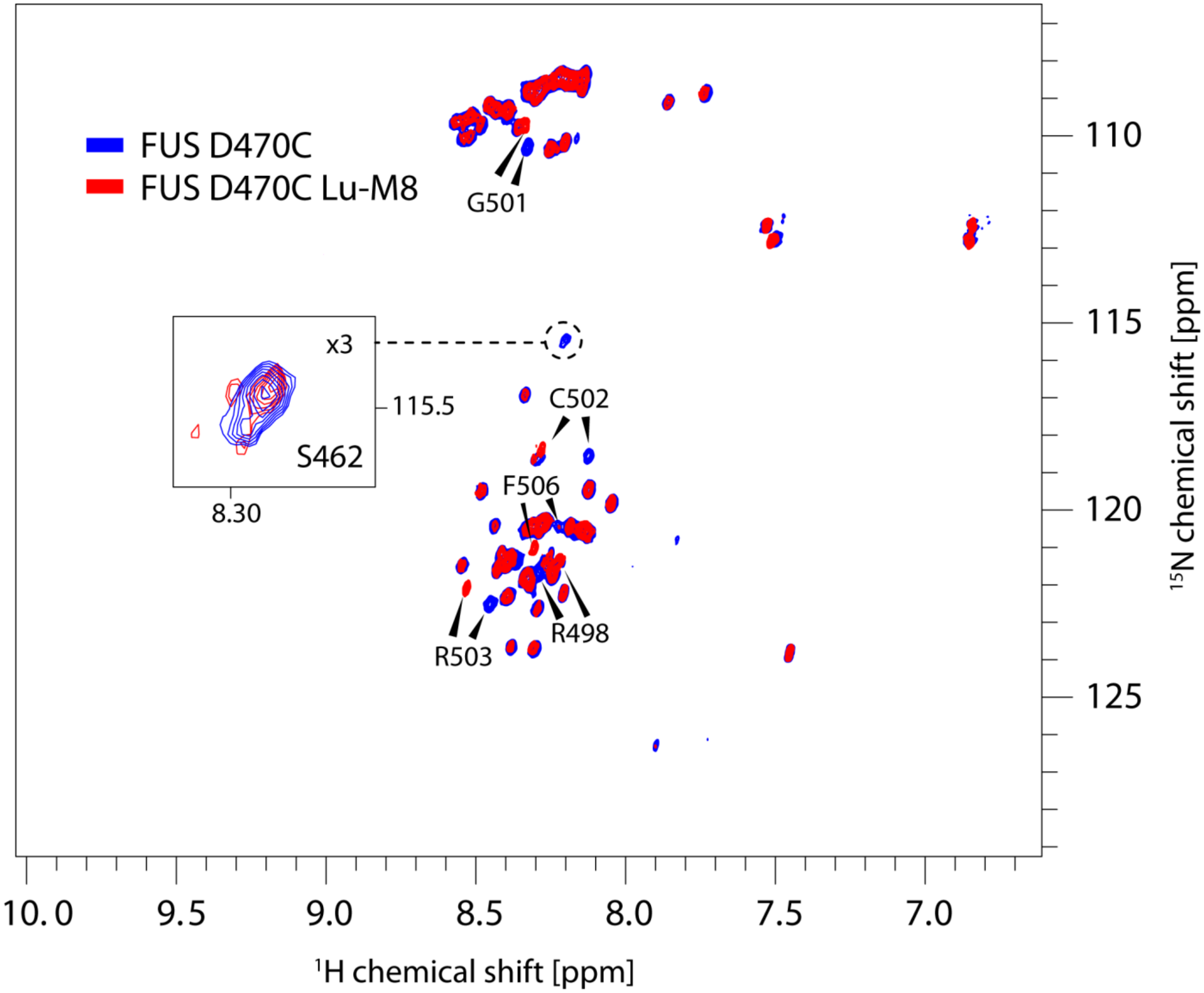

Figure S2. a) Overlay of $^{1}H^{15}N$ HSQC spectra of FUS 454-526 (D502C) before (blue) and after attachment of Lu-M8 (red). Shifting resonances were assigned and residues with substantial chemical shift changes are labeled. Only neighboring resonances are affected by the attachment of the Lu-M8 tag. The position of residue S462 is not altered but the overlay is only visible in the enlargement. b) Overlay of a histogram of ^{1}H chemical shift changes after the introduction of the Lu-M8 tag (blue) and the Yb-M8 tag (magenta) at position 470.

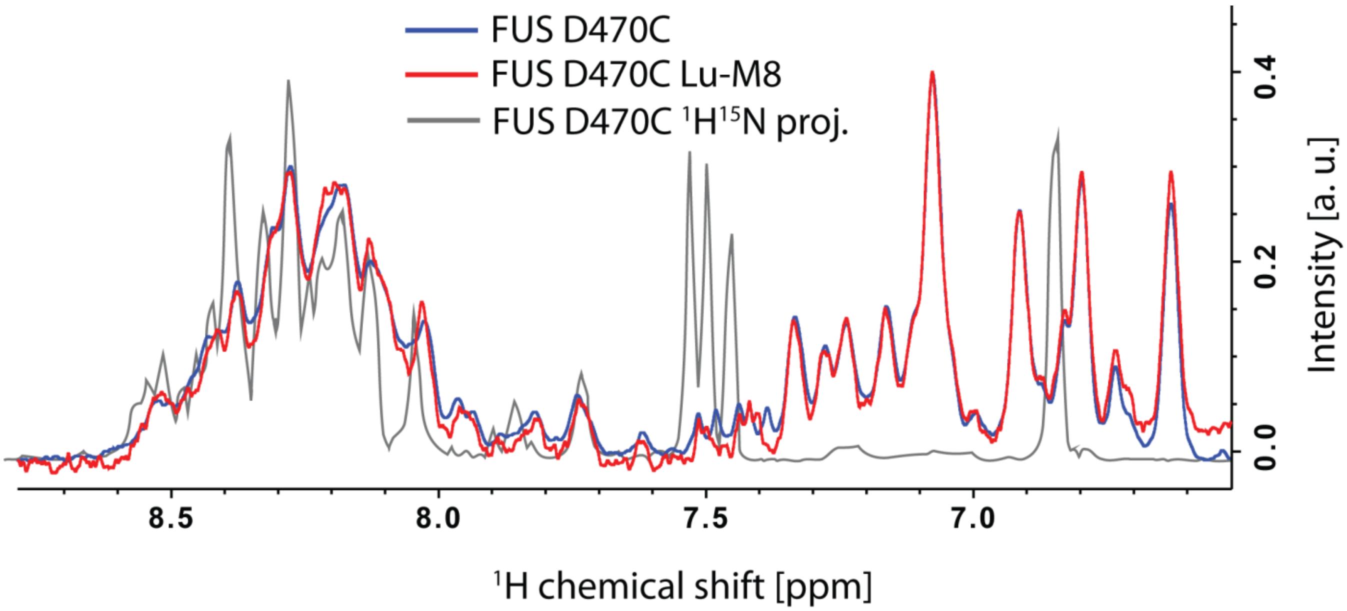

Figure S3. ^{1}H NMR spectrum of the D470C mutant before (blue) and after diamagnetic Lu-M8 labeling (red). In grey, the ^{1}H^{15}N projection is indicating the nitrogen bound protons of the untagged species. No changes in chemical shift can be observed for the aromatic residues.

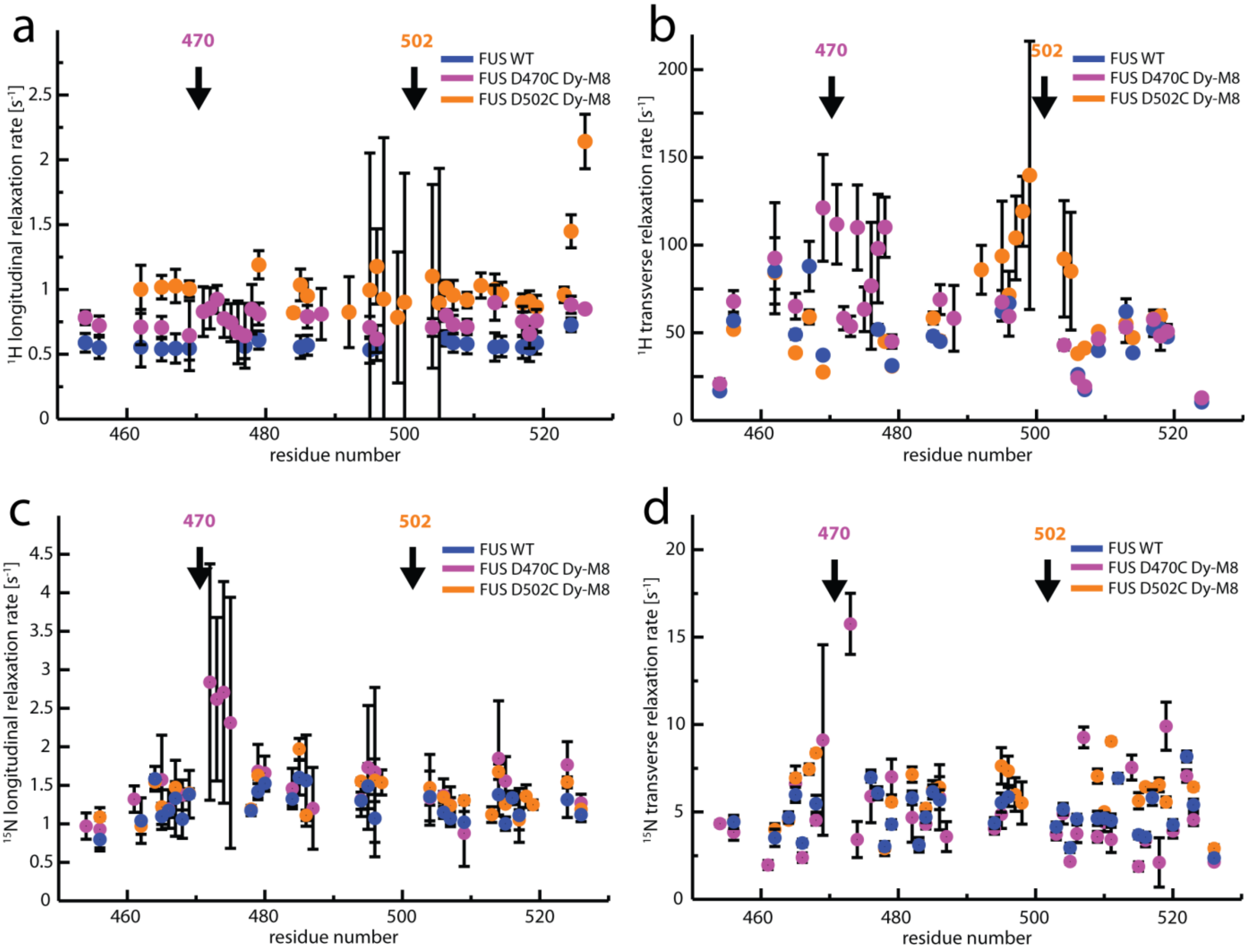

Figure S4. (a) Longitudinal (R_1) and (b) transverse (R_2) relaxation rates of amide protons of FUS(454-526) (blue) and of the Yb-M8 labeled D470C (pink) and D502C (orange) mutants, respectively. Elevated relaxation rates are found in the proximity of the label. The ^{1}H-R_1s of the Yb-M8 samples are generally higher than the WT reference. The ninth coordination site of the tag can directly bind water and modifies its R1 rate, which contributes to the protein magnetization build-up through chemical exchange.[10] This arises probably from a PRE effect of the Yb-M8 tag through chemical exchange of (relaxed) water protons with amide protons[10]. Indeed we noticed a slight sigmoidal shape in the ^{1}H-R_1 exponential recovery curves. Nevertheless, the data has been fit with a mono-exponential function to give an approximate value of the rates. Nitrogen R_1 (c) and R_2 (d) are shown in the lower panel. Peaks close to the tagging site show higher relaxation rates and values are not shown due to large errors. Together with the ^{15}N{^{1}H} heteronuclear NOE data (e), the relaxation data clearly show that introduction of the tag does not significantly change the dynamics of the tagged protein and that no interactions of the tag to remote sites can be observed. The data analysis was hampered by heavy overlap of a number of peaks. Resonances with clearly overlapped peaks and bi-exponential intensity decay have been removed.

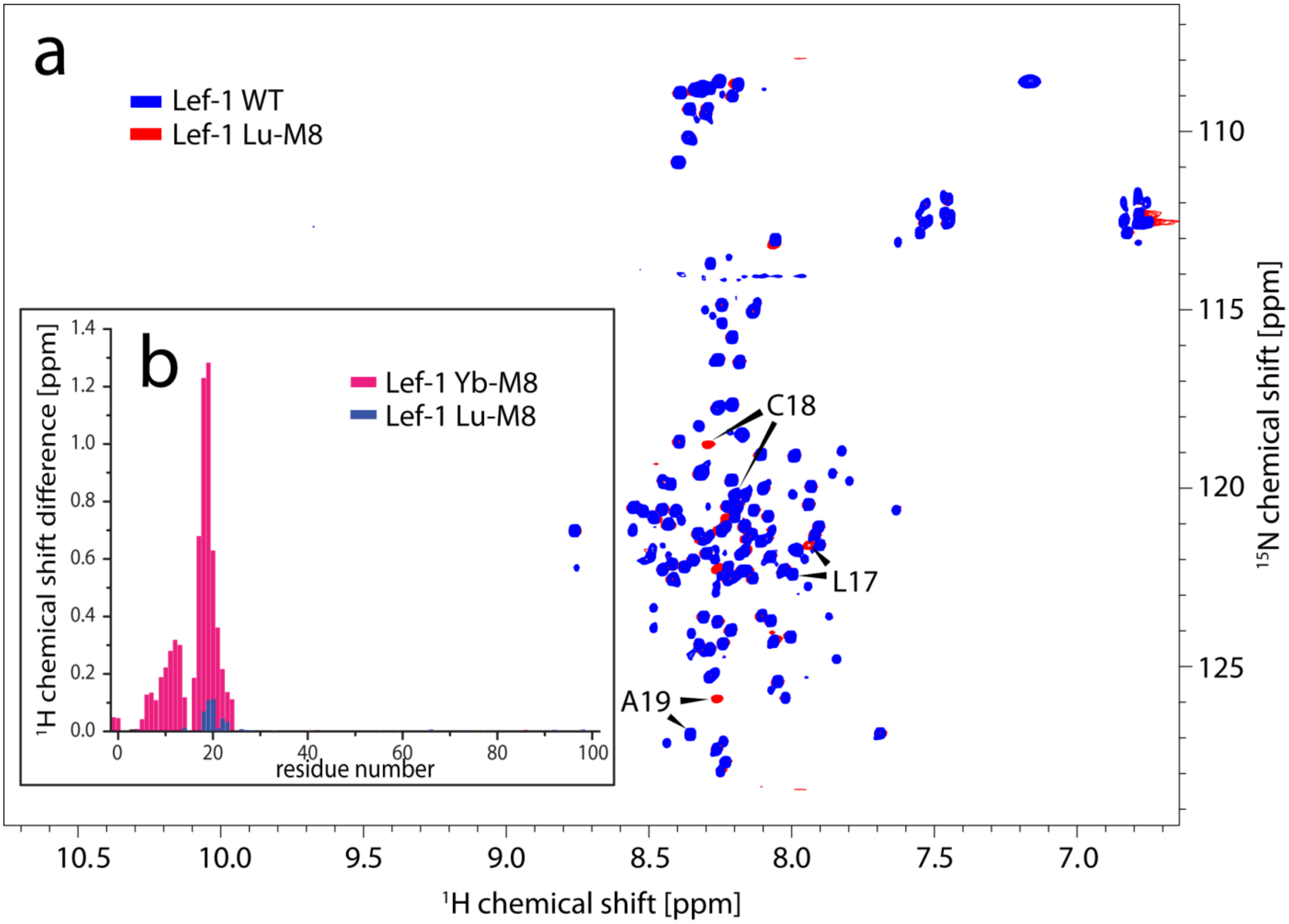

Figure S5. Overlay of $^{1}H^{15}N$ HSQC spectra of Lef-1 (1-101) before (blue) and after attachment of Lu-M8 (red). Shifting resonances were assigned and residues with substantial chemical shift changes are labeled. Only neighboring resonances are affected by the attachment of the Lu-M8 tag. b) Overlay of a histogram of ^{1}H chemical shift changes after the introduction of the Lu-M8 tag (blue) and the Yb-M8 tag (magenta).

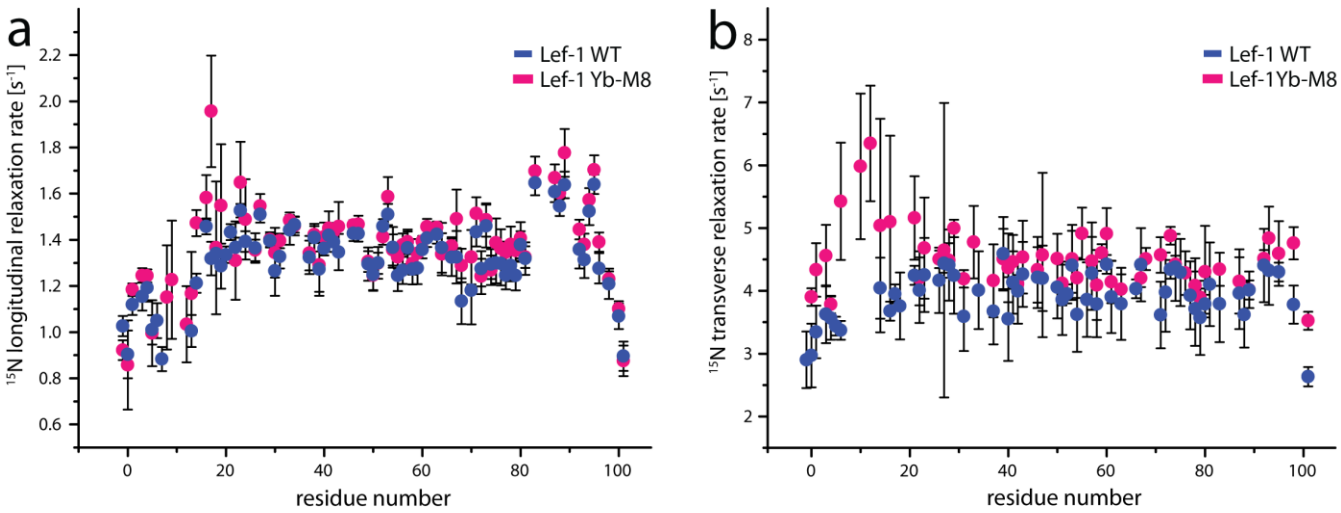

Figure S6. (a) Longitudinal and (b) transverse ^{15}N relaxation rates of Lef-1. The residues in the vicinity of the tagged Cys18 show a clear PRE influence but rates further away remain unaffected within the experimental error limits.

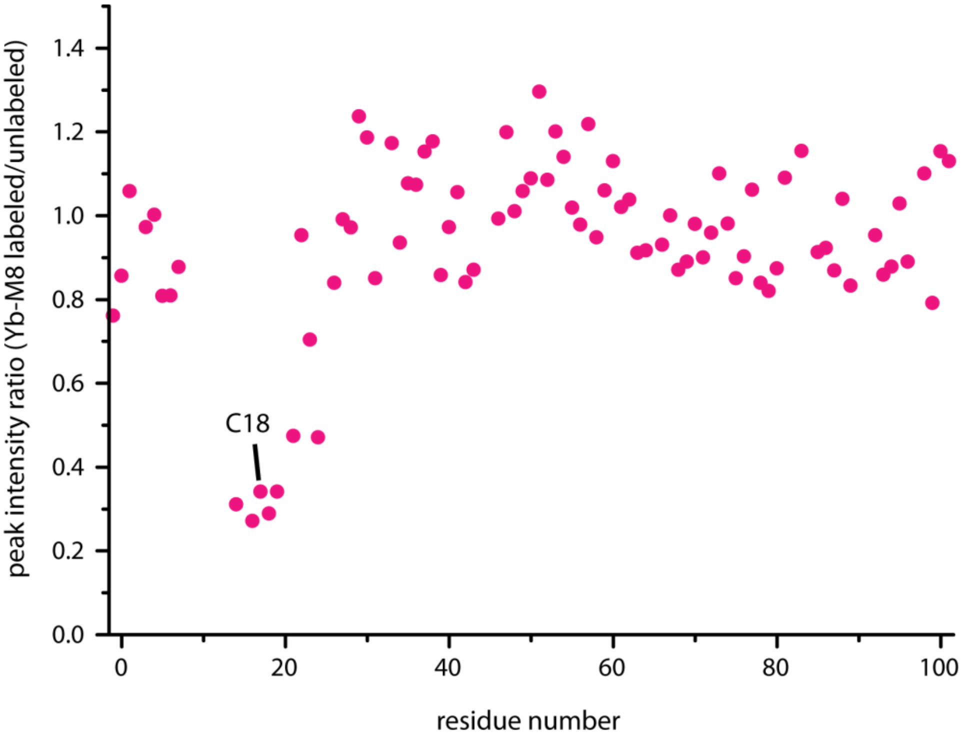

Figure S7. Peak intensity ratios of $^{1}H^{15}N$-HSQC spectra of Lef-1 labeled with Yb-M8 and the unlabeled reference. The position of label attachment is marked. The PRE effect of the introduced lanthanide leads to an intensity reduction of about 70%. Overlapping peaks (e. g. N-terminal the low complexity region) have been removed from the analysis. The spectra were acquired on two independent samples.

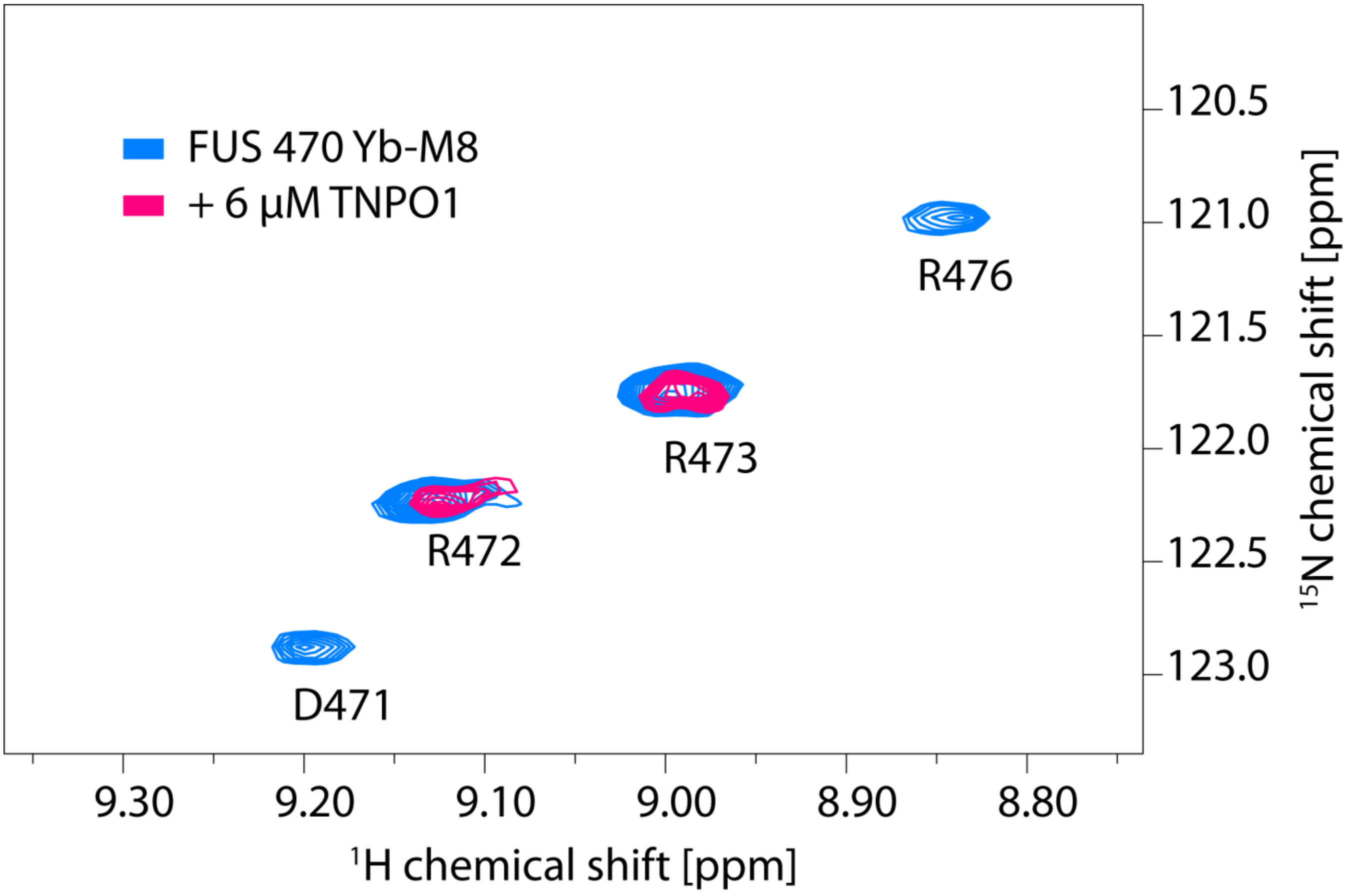

Figure S8. Overlay of $^{1}H^{15}N$ HSQC spectra of FUS (470 Yb-M8) before (blue) and after addition of 6 µM transportin-1, a monomeric 110 kDa nuclear import protein. The addition leads to a strong decrease of signal intensity which indicates binding of the region around residue 470 to transportin-1 in the intermediate exchange regime (note that after addition of 17 µM the peaks disappear completely). The data has been measured using the same parameters and magnetic fields as described above.
